# Solvatochromic Parameters of Four Amines in Propane-1,3-diol at 298.15 K

**DOI:** 10.3390/molecules30061213

**Published:** 2025-03-08

**Authors:** Maria-Luísa C. J. Moita, Ângela F. S. Santos, Miguel A. B. S. S. Correia, Isabel M. S. Lampreia

**Affiliations:** Centro de Química Estrutural, Departamento de Química e Bioquímica, Faculdade de Ciências, Institute of Molecular Sciences, Universidade de Lisboa, 1749-016 Lisboa, Portugal; afsantos@fc.ul.pt (Â.F.S.S.); miguel.soares.correia@outlook.com (M.A.B.S.S.C.); milampreia@fc.ul.pt (I.M.S.L.)

**Keywords:** solvatochromic parameters, propane-1,3-diol, alkoxyamines, alkanolamines

## Abstract

One of the most used methods for capturing acidic gases from the atmosphere is the use of amines that react with the acids and can later be recovered. The choice of amines that are most efficient in capturing has been the subject of several studies; however, the energy effort for their regeneration is also important. While the polarity of the solvent plays a critical role in determining which amines efficiently capture CO_2_, the heat capacity of the solvent is also a significant factor in the regeneration process. In this work, we present values for Reichardt’s $E_{T}^{N}\left(30\right)$ and Kamlet−Taft parameters, such as π* (dipolarity/polarizability), *α* (acidity), and *β* (basicity), for solutions of two alkanolamines and two alkoxyamines dissolved in propane-1,3-diol, at 298.15 K, a solvent with a lower heat capacity than water. In addition to the polarity characterization of the amines in that solvent, the aim of this study is to analyze the differences observed in the solvatochromic parameters when water is replaced by alcohol. The impact of this change on the values of those parameters for the binary amine + solvent solutions was assessed by calculating the transfer values, $\Delta_{\mathrm{transf}}\left(F_{i},x_{i}\right)$. Defined as, $\Delta_{\mathrm{transf}}\left(F_{i},x_{i}\right)=F_{i}\left(1,3\mathrm{PD}\right)-F_{i}\left(H_{2}O\right)$, these transfer values represent the difference in the parameters when the amines are transferred from water to alcohol. While the water medium is more favourable in terms of π* for CO_2_ capture, the alcohol medium appears to hold more promise in terms of *β*.

## 1. Introduction

Although the chemical-based proposals for the CO_2_ absorption mechanism by the amine-based CO_2_ capture method are based on the formation of carbamate and bicarbonate ions [1,2,3], its mechanism is not yet fully understood [4]. In fact, CO_2_ is a non-polar molecule with low solubility in polar mediums, such as aqueous amine solutions, and the absorption/adsorption process begins at the gas/liquid interface where intermediate short-living species formed are experimentally difficult to characterize.

The choice of different types of amines that have been revealed to be more efficient in the capture process has been the subject of many studies in recent years [5,6,7], showing that certain sterically hindered amines would be more efficient in this process [8]. However, considering the overall cost of the process and bearing in mind that throughout the process we will consider CO_2_ as the solute and the amine solution as the solvent, the subsequent regeneration of the amine is also an important step with high associated costs (between 60 and 70% of the total cost). One of the important characteristics of the solvents encompassing this last step of the process is its heat capacity; the lower it is, the lower energy costs are associated with it [6,9,10].

Recent studies combining experimental results on the absorption of CO_2_ by the well-studied 30% aqueous solution of 2-aminoethanol (MEA) with results from classical molecular dynamics (MD) showed the possibility of improving the CO_2_ capture process by modifying the polarity of the solvent to obtain more stable intermediate compounds in the interface of gas/liquid with better solubility in the solution of the amine, thus reducing the overall cost of the process [11].

The polarity of a solvent, which reflects its solvation capability, is influenced by the combined effects of all possible specific and non-specific solute–solvent interactions [12]. As a result, it serves as a crucial factor in the CO_2_ capture process. This concept has been extensively explored in numerous studies [12,13,14,15,16,17], including several outstanding reviews.

On the other hand, it was also recently found that there is a good correlation between the CO_2_ absorption capacity of a given amine and the Kamlet–Taft parameters *β* and π* of mixtures of a given organic solvent with water [3], being that the CO_2_ uptake is mainly governed by both the solvent basicity and dipolarity/polarizability. Based on these findings, the purpose of the present work is to characterize, in terms of the solvent polarity, four non-aqueous binary mixtures containing a dialcohol and one of two different alkanolamines or one of two different alkoxyamines.

Considering the characterization of the solvents regarding their ability to better adsorb the CO_2_ in the interface of gas/liquid and its release after capture, we aim to open other hints on suggesting the possible advantage of substituting water with a non-aqueous solvent, showing that the different solvatochromic parameters can be tuned out.

In this work, we determine the solvatochromic parameters, namely, Reichardt’s normalized parameter, $E_{T}^{N}\left(30\right)$; dipolarity/polarizability, π*; acidity, *α*; and basicity, *β*, in two secondary alkanolamines (one of them hindered) and two linear alkoxyamines in an organic solvent, propane-1,3-diol (1,3-PD), with a heat capacity of 2.31 kJ kg^−1^ K^−1^ [18] (nearly half that of the water 4.15 kJ kg^−1^ K^−1^), across the entire concentration range, and at 298.15 K. The differences observed in the amine-solvent parameters, when the amines are transferred from aqueous to the non-aqueous solvent, were also determined and analyzed.

## 2. Results and Discussion

Using the three molecular probes, 2,6-diphenyl-4-(2,4,6-triphenylpyridinium-1-yl) phenolate, Reichardt’s betaine, RB(30); 4-amino-nitrobenzene, 4-NA; and 4-(dimethylamino)-nitrobenzene, NN-4-NA, experimental wavelengths across the whole composition range of the binary liquid mixtures: {propane-1,3-diol + 2-(ethylamino)ethanol (EEA), or 2-(isopropylamino)ethanol (IPAE), or 3-ethoxypropan-1-amine (EPA), or 3-butoxypropan-1-amine (BPA)}, were obtained at 298.15 K. Values are presented in the Supplementary Information (SI), Table S1.

The solvent parameter $E_{T}\left(30\right)$ was calculated using Equation (1) [12,19,20]:

(1) $$\frac{E_{T}\left(30\;\right)}{\mathrm{kcal}\cdot {\mathrm{mol}}^{-1}}\;=N_{A}hc{\tilde{\nu }}_{\mathrm{RBprobe}}=2.8591\times 10^{-3}\left(\frac{{\tilde{\nu }}_{\mathrm{RBprobe}}}{{\mathrm{cm}}^{-1}}\right)$$

where *N*_A_ is the Avogadro number, *h* is the Planck constant, *c* is the speed of light in the vacuum, and ${\tilde{\nu }}_{\mathrm{RBprobe}}$ is the wavenumber of the RB probe. $E_{T}\left(30\right)$ was normalized to give a value of 0 for tetramethylsilane (TMS) and 1 for water according to Equation (2) [12,19,20].

(2) $$E_{T}^{N}\left(30\right)=\frac{E_{T}{\left(30\right)}_{\mathrm{solvent}}-30.7}{32.4}$$

The dipolarity/polarizability parameter, $\pi^{*}$, was calculated from Equation (3) to give a value of 0 for cyclohexane and 1 for dimethylsulfoxide [12,21]. ${\tilde{\nu }}_{\mathrm{NN}-4-\mathrm{NA}}$ is the wavenumber of the NN-4-NA probe.

(3) $$\pi^{*}=\frac{{\tilde{\nu }}_{\mathrm{NN}-4-\mathrm{NA}}-28.18}{-3.52}$$

The acidity parameter, *α*, was calculated using Equation (4), giving a value of 1 for methanol [12,21].

(4) $$\alpha =\frac{1.318{\tilde{\nu }}_{\mathrm{NN}-4-\mathrm{NA}}-47.7+{\tilde{\nu }}_{\mathrm{RB}\left(30\right)}}{5.47}$$

The basicity parameter, *β*, was estimated to give a value of 1 for hexamethylphosphoramide and is given by Equation (5) [12,21]. ${\tilde{\nu }}_{4-\mathrm{NA}}$ is the wavenumber of the 4-NA probe.

(5) $$\beta =\frac{0.9841{\tilde{\nu }}_{\mathrm{NN}-4-\mathrm{NA}}+3.49-{\tilde{\nu }}_{4-\mathrm{NA}}}{2.759}$$

**Figure 1:**
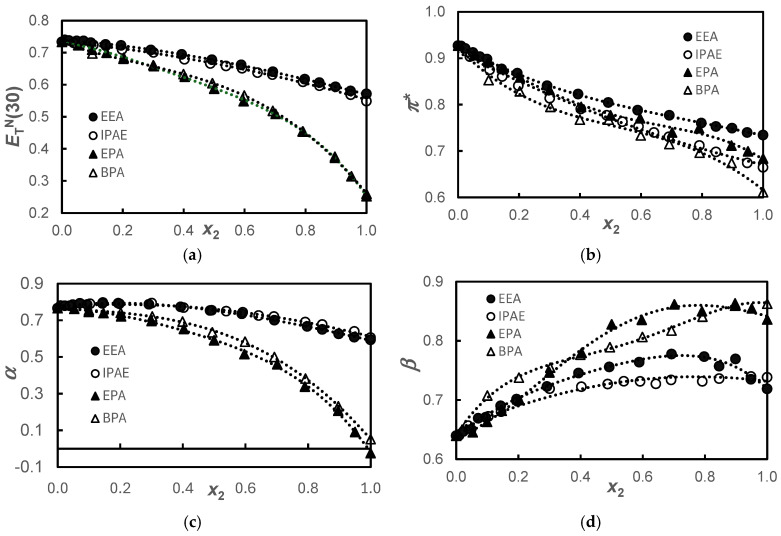
Solvatochromic parameters, $E_{T}^{N}\left(30\right)\;\left(a\right)$; π* (**b**); *α* (**c**); and *β* (**d**), for the {propane-1,3-diol+amine} systems (●), EEA; (**ο**), IPAE; (▲), EPA; and (**Δ**), BPA at *T* = 298.15 K. The dotted lines were drawn to guide the eyes adjusting polynomial functions.

Results for $E_{T}^{N}\left(30\right)$, $\pi^{*}$, *α*, and *β* are shown in Table 1 and displayed in Figure 1.

Since the four solvatochromic parameters of the pure amphiphiles were previously published [22,23], in Table 2, we report their values for comparison. The displayed uncertainty values show a good agreement within the mutual uncertainty for all the parameters.

Figure 1 shows similar behaviors of $E_{T}^{N}\left(30\right)$ and *α* for the alkanolamine and alkoxyamine pairs. Knowing that the $E_{T}^{N}\left(30\right)$ parameter measures both the dipolarity/polarizability, π*, and acidity, *α*, of the solvent, it can be observed that in these systems, the $E_{T}^{N}\left(30\right)$ parameter predominantly reflects the acidity of the solvent since the behavior of π* does not show that same pairing. This dichotomy of *α* and π* behavior is explained because, regarding acidity, we have two very different types of compounds, with pure basic alkoxyamines having acidity very close to zero and alkanolamines being rather more acidic. On the other hand, regarding the dipolarity/polarizability, we found that the size of the amine molecules is the factor that governs the observed behavior.

Regarding basicity, as previously mentioned, alkoxyamines are inherently more basic than alkanolamines. In the same class of compounds, the fact that the pure compound with the longer chain is more basic than the pure compound with the shorter chain could be justified by the inductive effect that would exist in molecules with longer carbon chains, displacing the negative charge towards the more electronegative atoms, -O- in the alkoxyamines and -NH- in the alkanolamines. Also, synergistic effects are observed for the systems of the smallest molecules in the concentrated composition ranges 0.4 < *x*_2_ < 0.9 and 0.7 < *x*_2_ < 0.9, for EEA and EPA, respectively. This peculiar behavior had already been observed and interpreted by us in the systems H_2_O + EEA [22] and H_2_O + EPA [23] as being the result of the formation of the stable entities EPA·H_2_O or EEA·H_2_O as water is added starting from the pure amphiphile. It is believed that, in the same way, the stable entities EEA·1,3-PD or EPA·1,3-PD would be formed. This phenomenon has been previously observed in earlier studies [14,16,17], where the role of hydrogen bonding in enhancing polarity compared to the pure components was thoroughly discussed. In the present study, however, this effect is only evident in basicity.

**Figure 2:**
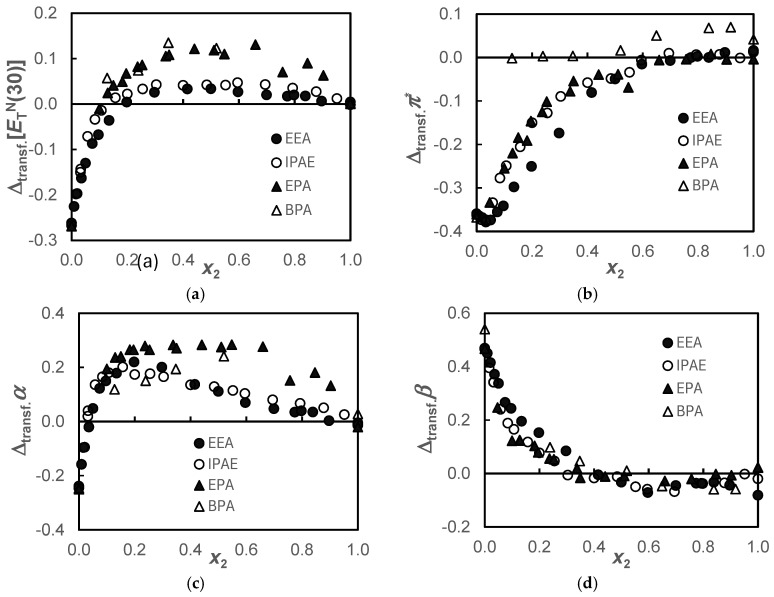
Transfer values, $\Delta_{\mathrm{transf}}\left(F_{i},x_{i}\right)=F_{i}\left(1,3–\mathrm{PD}\right)-F_{i}\left(H_{2}O\right)$, of the solvatochromic parameters, $E_{T}^{N}\left(30\right)$ (**a**); π* (**b**); *α* (**c**); and *β* (**d**), for the {propane-1,3-diol+amine} systems when the amines are transferred from water to the alcohol, at *T* = 298.15 K.

As stated in the introduction, recent studies, with the aim of outlining alternatives to the use of aqueous solvents to optimize the cost-benefit of the carbon capture and storage process (CCS), indicate that non-aqueous solvents may present advantages. In this work, we chose a dialcohol, namely, 1,3-PD, with the aim of forming non-aqueous solutions with two secondary amines, one of which is hindered, and two primary amines and evaluating the change in the characteristics of the solvent in terms of its solvatochromic parameters when water is replaced by this alcohol. A convenient way to achieve this goal is to calculate the transfer values of those parameters when the amines are transferred from water to alcohol. The transfer function, Δ_transf_(*F_i_,x_i_*), is defined by Equation (6) and the results are presented in Table S2 and Figure 2:

(6) $$\Delta_{\mathrm{transf}}\left(F_{i},x_{i}\right)=F_{i}\left(1,3–\mathrm{PD}\right)-F_{i}\left(H_{2}O\right)$$

$F_{i}\left(1,3–\mathrm{PD}\right)$ and $F_{i}\left(H_{2}O\right)$ are the values of each solvatochromic parameter, $E_{T}^{N}\left(30\right)$, π*, *α*, or *β*, at the same composition, *x_i_*, of the amine in 1,3-PD and in water, respectively.

The pairing of the $E_{T}^{N}\left(30\right)$ behavior observed in Figure 1a for the secondary amines EEA and IPAE and the primary amines EPA and BPA is also verified in the transfer function, $\Delta_{\mathrm{transf}}E_{T}^{N}\left(30\right)$, depicted in Figure 2a. This transfer function presents negative values up to *x*_2_ ≈ 0.2, becoming positive from this molar fraction onward, but more positive ones in primary amines. Since $E_{T}^{N}\left(30\right)$ is a mixed parameter, simultaneously measuring dipolarity/polarizability, π*, and acidity, *α*, these parameters will be analyzed below. In Figure 2c, the same pairing described for the $E_{T}^{N}\left(30\right)$ function is observed for α, showing that this last function predominantly reflects the acidity of the solvent.

Regarding the $\Delta_{\mathrm{transf}}\pi^{*}$, Figure 2b, the marked negative π* difference between alcohol and water attenuates as the amine is added, becoming practically null from *x*_2_ ≈ 0.6 for the EEA, IPAE, and EPA systems and from *x*_2_ ≈ 0.1 for the BPA system. However, a minimum is observed in the case of secondary amines for *x*_2_ ≈ 0.03. In the BPA system, the values are slightly positive, although they are within mutual uncertainty.

Concerning the $\Delta_{\mathrm{transf}}\beta$ function in all systems, there is a zone of greater basicity of the amine in alcohol compared to that of the amine in water for a range of mole fractions 0 ≤ *x*_2_ ≤ 0.4. This fact indicates that the 1,3-PD + amine solvent would be more promising in capturing CO_2_ in the range of molar fractions indicated above. Figure 2c shows that despite water being much more acidic than 1,3-PD, giving negative values for the transfer property, as amine is added, they rapidly became positive beyond *x*_2_ > 0.03. Curiously, between this composition and *x*_2_ = 0.4, there is also a higher basicity (Figure 2d) of all amines in alcohol than in water. This is probably due to the coexisting hydrogen bond acceptor and hydrogen bond donor capabilities of the solvent in the alcohol. This effect is more pronounced in the alkoxyamine systems.

## 3. Experimental

### 3.1. Materials

All the amines and the propane-1,3-diol were reagent grade and were used as received without further purification. Table 3 presents the characterization of the chemicals used.

### 3.2. Methods

All solutions were prepared by mass using a Kern AEJ balance (Kern & Sohn, Balingen, Germany) with a resolution of 0.00001 g. Airtight designed flasks provided with Teflon stoppers were used to avoid evaporation and air contamination. Different total volumes of solution were used, attending to the minimization of the vapor phase. Buoyancy corrections were applied. Standard combined uncertainties in the calculated mole fractions were found to be less than 0.0005.

UV−vis Spectroscopy: Wavelengths of the molecular probes in the solutions prepared were performed with a double-beam UV-1800 Shimazu spectrometer (Shimazu, Kyoto, Japan) with a resolution of 0.1 nm. The path length of the cell used was 1 cm. The temperature was kept constant at (298.15 ± 0.10) K. The amount of each dye, added directly to the cell, was evaluated to obtain absorbance values ranging from 0.5 to 1 to avoid the formation of aggregates. The spectra were recorded 3 to 5 times and the wavelength corresponding to the peak of maximum absorbance, *λ*_max_, was obtained from the average. The uncertainty of the *λ*_max_ was found to be 1 nm. The wavenumbers of maximum absorbance of each dye, ${\tilde{\nu }}_{\mathrm{dye}}$, expressed in kilo Kaiser (1 kK = 1000 cm^−1^), were used to calculate the solvatochromic parameters.

## 4. Conclusions

In this study, the polarity of four binary mixtures was characterized at 298.15 K. These mixtures consisted of a dialcohol, propane-1,3-diol, combined with either two secondary amines—2-(ethylamino)ethanol, EEA, or the sterically hindered 2-(isopropylamino)ethanol, IPAE—or two primary amines—3-ethoxy-1-propylamine, EPA, or 3-butoxy-1-propylamine, BPA. Polarity measurements were conducted using three molecular probes: 2,6-diphenyl-4-(2,4,6-triphenylpyridinium-1-yl) phenolate (Reichardt’s betaine, RB(30)); 4-amino-nitrobenzene (4-NA); and 4-(dimethylamino)-nitrobenzene (NN-4-NA). This study determined values for Reichardt’s $E_{T}^{N}\left(30\right)$ as well as Kamlet–Taft parameters, including π* (dipolarity/polarizability), *α* (acidity), and *β* (basicity).

Similar trends for $E_{T}^{N}\left(30\right)$ and *α* in alkanolamine and alkoxyamine pairs were observed. Since $E_{T}^{N}\left(30\right)$ reflects both dipolarity/polarizability (π*) and acidity (*α*), the acidity contribution is the dominant factor as π* does not follow the same pattern.

Regarding basicity within each class, longer-chain compounds are more basic due to the inductive effect, which shifts the negative charge toward more electronegative atoms (-O- in alkoxyamines and -NH- in alkanolamines). Additionally, synergistic effects were observed in concentrated mixtures of the small EEA and EPA molecules, likely due to the formation of stable entities, such as EEA·1,3-PD or EPA·1,3-PD, similar to previously observed EPA·H_2_O and EEA·H_2_O formations, mainly due to H-bonding.

This study investigates the potential benefits of using non-aqueous solvents, specifically 1,3-propanediol (1,3-PD), as an alternative to water-based solvents for carbon capture. By analyzing solvatochromic parameters, the research evaluates how replacing water with 1,3-PD influences solvent properties. The transfer values of solvent polarity parameters, when amines are transferred from water to alcohol, suggest that in the presence of amines, 1,3-PD medium exhibits greater basicity than water medium, particularly within the mole fraction range of 0 ≤ x_2_ ≤ 0.40, making it a more promising candidate for CO_2_ capture.

## Figures and Tables

**Table 1:** Solvatochromic parameters for {1,3-PD (1) + EEA, or IPAE, or EPA, or BPA (2)} binary mixtures at *T*/K = 298.15 and *P*/MPa = 0.1 **^a^**.

1,3-PD (1) + EEA (2) ^b^					1,3-PD (1) + IPAE (2) ^c^				
*x*_2_	$E_{T}^{N}\left(30\right)$	π*	*α*	*β*	*x_2_*	$E_{T}^{N}\left(30\right)$	π*	*α*	*β*
0.0000	0.733	0.927	0.766	0.64	0.0000	0.733	0.927	0.766	0.64
0.0099	0.740	0.926	0.779	0.64	0.0388	0.730	0.903	0.779	0.66
0.0233	0.737	0.921	0.779	0.65	0.1037	0.724	0.875	0.790	0.67
0.0486	0.737	0.912	0.785	0.65	0.1447	0.721	0.861	0.796	0.68
0.0713	0.737	0.903	0.792	0.67	0.1975	0.710	0.841	0.790	0.70
0.0949	0.731	0.898	0.786	0.67	0.3007	0.700	0.814	0.794	0.72
0.1432	0.725	0.877	0.792	0.69	0.4026	0.679	0.791	0.769	0.72
0.1942	0.722	0.868	0.793	0.70	0.4875	0.666	0.777	0.753	0.73
0.2919	0.708	0.841	0.787	0.72	0.5389	0.658	0.763	0.749	0.73
0.3933	0.694	0.823	0.773	0.75	0.5917	0.651	0.753	0.743	0.73
0.4924	0.678	0.804	0.754	0.76	0.6424	0.637	0.740	0.726	0.73
0.5889	0.662	0.788	0.735	0.76	0.6912	0.631	0.731	0.720	0.73
0.6926	0.640	0.777	0.700	0.78	0.7913	0.609	0.712	0.690	0.73
0.7975	0.617	0.760	0.666	0.77	0.8457	0.597	0.698	0.677	0.74
0.8455	0.606	0.753	0.650	0.76	0.9483	0.569	0.674	0.639	0.74
0.8983	0.593	0.749	0.626	0.77	1.0000	0.549	0.665	0.605	0.74
0.9479	0.581	0.740	0.608	0.73					
1.0000	0.571	0.734	0.593	0.72					
**1,3-PD (1) + EPA (2) ^d^**					**1,3-PD (1) + BPA (2) ^e^**				
***x*_2_**	$E_{T}^{N}\left(30\right)$	π*	***α***	***β***	***x*_2_**	$E_{T}^{N}\left(30\right)$	π*	***α***	***β***
0.0000	0.733	0.927	0.766	0.64	0.0000	0.733	0.927	0.766	0.64
0.0533	0.723	0.906	0.762	0.65	0.0999	0.697	0.853	0.754	0.71
0.0993	0.709	0.892	0.746	0.66	0.2018	0.681	0.829	0.741	0.74
0.1466	0.700	0.877	0.739	0.68	0.3023	0.658	0.795	0.721	0.75
0.2031	0.684	0.859	0.722	0.70	0.3992	0.633	0.768	0.693	0.78
0.3000	0.661	0.832	0.696	0.75	0.4941	0.605	0.768	0.634	0.79
0.4033	0.625	0.795	0.653	0.78	0.5987	0.566	0.734	0.583	0.81
0.4998	0.587	0.777	0.591	0.83	0.6923	0.518	0.715	0.500	0.82
0.5965	0.549	0.771	0.516	0.84	0.7917	0.454	0.696	0.382	0.84
0.7015	0.509	0.740	0.461	0.86	0.8966	0.372	0.674	0.231	0.86
0.7899	0.454	0.749	0.338	0.85	1.0000	0.260	0.611	0.052	0.86
0.8962	0.375	0.712	0.207	0.86					
0.9503	0.315	0.699	0.091	0.85					
1.0000	0.252	0.684	−0.025	0.84					

^a^ Standard uncertainties, *u*, are *u*(*T*)/K = 0.05, *u*_c_(*x*_2_) = 0.0005, and *u*(*P*)/MPa = 0.02. ^b^ *u*[${\;E}_{T}^{N}$(30)] = 0.005; *u*(π*) = 0.03; *u*(α) = 0.03; and *u*(β) = 0.08. ^c,d^ *u*[${\;E}_{T}^{N}$(30)] = 0.005; *u*(π*) = 0.03; *u*(α) = 0.03; and *u*(β) = 0.06. ^e^ *u*[${\;E}_{T}^{N}$(30)] = 0.005; *u*(π*) = 0.05; *u*(α) = 0.03; and *u*(β) = 0.06.

**Table 2:** Solvatochromic parameters for the pure amines, EEA, IPAE, EPA, or BPA, at *T*/K = 298.15 and *P*/MPa = 0.1 **^a^**.

	EEA		IPAE		EPA		BPA	
	This Work	Lit. ^a^	This Work	Lit. ^a^	This Work	Lit. ^b^	This Work	Lit. ^b^
$E_{T}^{N}$(30)	0.571	0.565	0.549	0.551	0.252	0.253	0.26	-
	(0.005)	(0.008)	(0.005)	(0.005)	(0.005)	(0.005)	(0.005)	
π*	0.734	0.718	0.665	0.652	0.684	0.684	0.611	0.574
	(0.03)	(0.05)	(0.03)	(0.03)	(0.03)	(0.03)	(0.05)	(0.03)
*α*	0.593	0.595	0.605	0.621	−0.025	0.000	0.052	-
	(0.03)	(0.05)	(0.03)	(0.03)	(0.03)	(0.03)	(0.03)	
*β*	0.72	0.80	0.74	0.76	0.84	0.82	0.86	0.84
	(0.08)	(0.08)	(0.06)	(0.06)	(0.06)	(0.06)	(0.06)	(0.06)

^a^ [22]; ^b^ [23].

**Table 3:** Characteristics of the chemicals used.

Chemicals	Acronym	Molecular Structure	Chemical Formula Molar Mass/g·mol^−1^ CAS Number	Source Purity/Mass Fraction
2-(ethylamino) ethanol	EEA	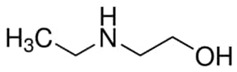	C_4_H_11_NO 89.13624 110-73-6	ACROS-ORGANICS (Geel, Belgium) >0.98 (value supplied by the manufacturer); used as received.
2-(isoproylamino) ethanol	IPAE	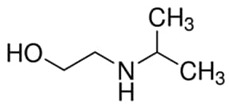	C_5_H_13_NO 103.1629 109-56-8	TCI-Europe (Zwijndrecht, Belgium) >0.99 (value supplied by the manufacturer); used as received.
3-ethoxy-1-propylamine	EPA	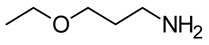	C_5_H_13_NO 103.1629 6291-85-6	Sigma-Aldrich (Saint Louis, MO, USA) >0.99 (value supplied by the manufacturer); used as received.
3-butoxy-1-propylamine	BPA	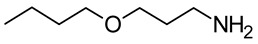	C_7_H_17_NO 131.2160 16499-88-0	Sigma-Aldrich (Saint Louis, MO, USA) >0.99 (value supplied by the manufacturer); used as received.
propane-1,3-diol	1,3-PD	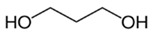	C_3_H_8_O_2_ 76.09442 504-63-2	Merck (San Jose, CA, USA) > 0.98 (value supplied by the manufacturer); used as received.
2,6-diphenyl-4-(2,4,6-triphenylpyridinium-1-yl)phenolate Reichardt’s Betaine 30	RB(30)	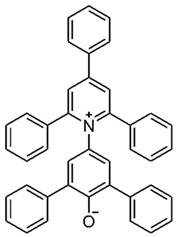	C_41_H_29_NO 551.68 10081-39-7	Sigma Aldrich (Saint Louis, MO, USA) >0.90 (used as received)
4-amino-nitrobenzene	4-NA	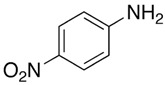	C_29_H_19_Cl_2_N 138.12 100-01-6	Sigma Aldrich (Saint Louis, MO, USA) >0.99 (used as received)
4-(dimethylamino)-nitrobenzene	NN-4-NA	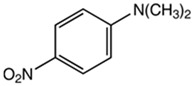	C_8_H_10_N_2_O_2_ 166.07 100-23-2	TCI (Pleasant Prairie, WI, USA) >0.99 (used as received)

## Data Availability

The original contributions presented in this study are included in the article/Supplementary Materials. Further inquiries can be directed to the corresponding author.

## Supplementary Materials

The following supporting information can be downloaded at: https://www.mdpi.com/article/10.3390/molecules30061213/s1, Table S1: Wavelengths of maximum absorbances, λ/nm, of the molecular probes: Reichardt’s Betaine, RB(30), 4-amino-nitrobenzene, 4-NA, and 4-(dimethylamino)-nitrobenzene, NN-4-NA, in the binary liquid mixtures: {propane-1,3-diol + 2-(ethylamino)ethanol (EEA) or 2-(isopropylamino)ethanol (IPAE) or 3-ethoxypropan-1-amine (EPA) or 3-butoxypropan-1-amine (BPA)}, at *T*/K = 298.15 and P/MPa = 0.1. Table S2: Transfer functions, $\Delta_{\mathrm{transf}}\left(F_{i},x_{i}\right)=F_{i}\left(1,3–\mathrm{PD}\right)-F_{i}\left(H_{2}O\right)$ of the amines-solvent parameters $E_{T}^{N}\left(30\right)$; dipolarity/polarizability, *π**; acidity, *α* and basicity, *β*, when the amines are transferred from the aqueous to the non-aqueous solvent, at *T*/K =298.15 and P/MPa = 0.1.
