# Supplementary material for: Solvatochromic Parameters of Four Amines in Propane-1,3-diol at 298.15 K
Source: Molecules. 2025 Mar 8;30(6):1213. doi: 10.3390/molecules30061213 (PMC11946820; doi:10.3390/molecules30061213)
Supplement: Supplementary file 1 [file molecules-30-01213-s001.zip › molecules-3478431-supplementary.pdf]

Supporting Information:

Solvatochromic parameters of four amines in propane-1,3-diol at 298.15 K

Maria-Luísa C.J. Moita*, Ângela F.S. Santos, Miguel A.B.S.S. Correia, Isabel M.S. Lampreia

Table S1.

Wavelengths of maximum absorbances, λ/nm, of the molecular probes: Reichardt's Betaine, RB(30), 4-amino-nitrobenzene, 4-NA, and 4-(dimethylamino)-nitrobenzene, NN-4-NA, in the binary liquid mixtures: {propane-1,3-diol + 2-(ethylamino)ethanol (EEA) or 2-(isopropylamino)ethanol (IPAE) or 3-ethoxypropan-1-amine (EPA) or 3-butoxypropan-1-amine (BPA)}, at *T*/K =298.15 and P/MPa = 0.1.

λ/nm							
1,3-PD (1) + EEA (2)				1,3-PD (1) + IPAE (2)			
x_2	RB(30)	4-NA	NN-4-NA	x_2	RB(30)	4-NA	NN-4-NA
0.0000	525.0	381.0	401.3	0.0000	525.0	381.0	401.3
0.0099	523.0	381.0	401.3	0.0388	526.0	380.5	400.0
0.0233	523.7	381.0	401.0	0.1037	528.0	379.7	398.4
0.0486	524.0	380.7	400.5	0.1447	528.9	379.3	397.6
0.0713	524.0	381.0	400.0	0.1975	532.5	379.0	396.5
0.0949	525.7	380.8	399.7	0.3007	535.5	378.5	395.0
0.1432	527.5	380.5	398.5	0.4026	542.5	377.5	393.8
0.1942	528.5	380.5	398.0	0.4875	547.0	377.0	393.0
0.2919	533.0	380.0	396.5	0.5389	549.5	376.5	392.3
0.3933	537.5	380.0	395.5	0.5917	552.0	376.0	391.7
0.4924	543.0	379.5	394.5	0.6424	556.8	375.2	391.0
0.5889	548.4	379.0	393.6	0.6912	559.0	375.0	390.5
0.6926	555.8	379.0	393.0	0.7913	567.0	374.0	389.5
0.7975	564.0	378.0	392.1	0.8457	571.5	373.5	388.8
0.8455	567.9	377.0	391.7	0.9483	582.0	372.5	387.5
0.8983	572.8	377.3	391.5	1.0000	589.8	372.0	387.0
0.9479	577.5	375.5	391.0				
1.0000	581.0	374.6	390.7				

λ/nm							
1,3-PD (1) + EPA (2)				1,3-PD (1) + BPA (2)			
x_2	RB(30)	4-NA	NN-4-NA	x_2	RB(30)	4-NA	NN-4-NA
0.0000	525.0	381.0	401.3	0.0000	525.0	381.0	401.3
0.0533	528.2	380.2	400.2	0.0999	536.5	380.0	397.2
0.0993	532.7	380.2	399.3	0.2018	541.8	380.0	395.8
0.1466	535.7	380.2	398.5	0.3023	549.7	379.0	394.0
0.2031	540.8	380.0	397.5	0.3992	558.3	378.5	392.5
0.3000	548.7	380.5	396.0	0.4941	568.5	379.0	392.5
0.4033	561.2	380.0	394.0	0.5987	583.0	378.0	390.7
0.4998	574.9	381.0	393.0	0.6923	602.0	377.5	389.7
0.5965	589.8	381.0	392.7	0.7917	629.7	377.5	388.7
0.7015	605.7	380.5	392.0	0.8966	668.8	377.3	387.5
0.7899	629.5	380.5	391.5	1.0000	731.0	374.2	384.2
0.8962	667.0	379.0	389.5				
0.9503	699.2	378.2	388.8				
1.0000	735.5	376.7	388.0				

Table S2.

Transfer functions, $\Delta_{\text{transf}}(F_i, x_i) = F_i(1{,}3\text{–PD}) - F_i(\text{H}_2\text{O})$ of the amines-solvent parameters $E_T^N(30)$; dipolarity/polarizability, π^*; acidity, α and basicity, β, when the amines are transferred from the aqueous to the non-aqueous solvent, at T/K =298.15 and P/MPa = 0.1.

1,3-PD (1) + EEA (2)					1,3-PD (1) + IPAE (2)				
x_2	$\Delta_{\text{transf}}E_T^N(30)$	$\Delta_{\text{transf}}\pi^*$	$\Delta_{\text{transf}}\alpha$	$\Delta_{\text{transf}}\beta$	x_2	$\Delta_{\text{transf}}E_T^N(30)$	$\Delta_{\text{transf}}\pi^*$	$\Delta_{\text{transf}}\alpha$	$\Delta_{\text{transf}}\beta$
0.0000	-0.262	-0.359	-0.238	0.47	0.0000	-0.267	-0.362	-0.246	0.47
0.0095	-0.225	-0.365	-0.158	0.45	0.0164	-0.198	-0.373	-0.096	0.40
0.0206	-0.197	-0.369	-0.095	0.42	0.0320	-0.150	-0.377	0.019	0.34

0.0357	-0.163	-0.374	-0.020	0.37	0.0331	-0.143	-0.378	0.040	0.34
0.0505	-0.130	-0.374	0.048	0.34	0.0580	-0.071	-0.334	0.136	0.24
0.0746	-0.087	-0.355	0.122	0.27	0.0843	-0.034	-0.277	0.165	0.19
0.0966	-0.068	-0.342	0.150	0.24	0.1072	-0.014	-0.249	0.180	0.17
0.1348	-0.036	-0.298	0.179	0.20	0.1574	0.014	-0.205	0.202	0.12
0.1981	0.004	-0.250	0.220	0.15	0.2003	0.022	-0.150	0.174	0.08
0.2976	0.026	-0.174	0.201	0.08	0.2553	0.033	-0.128	0.177	0.05
0.4154	0.033	-0.081	0.137	0.00	0.3040	0.043	-0.090	0.165	-0.01
0.5000	0.034	-0.050	0.111	-0.03	0.4004	0.041	-0.058	0.135	-0.02
0.5969	0.027	-0.015	0.070	-0.07	0.4852	0.042	-0.049	0.129	-0.01
0.6993	0.020	-0.007	0.047	-0.05	0.5523	0.042	-0.034	0.115	-0.05
0.7740	0.017	0.000	0.035	-0.04	0.5953	0.047	-0.006	0.103	-0.06
0.7977	0.020	0.002	0.039	-0.04	0.6946	0.043	0.010	0.080	-0.07
0.8386	0.018	0.000	0.035	-0.03	0.7932	0.035	0.006	0.067	-0.04
0.8967	0.006	0.012	0.003	-0.04	0.8776	0.027	0.007	0.051	-0.04
1.0000	0.005	0.016	-0.005	-0.08	0.9518	0.012	-0.001	0.026	0.00
					1.0000	-0.002	0.012	-0.014	-0.02
**1,3-PD (1) + EPA (2)**					**1,3-PD (1) + BPA (2)**				
x_2	$\Delta_{\mathrm{transf}} E_{\mathrm{T}}^{\mathrm{N}}(30)$	$\Delta_{\mathrm{transf}} \pi^*$	$\Delta_{\mathrm{transf}} \alpha$	$\Delta_{\mathrm{transf}} \beta$	x_2	$\Delta_{\mathrm{transf}} E_{T}^{N}(30)$	$\Delta_{\mathrm{transf}} \pi^*$	$\Delta_{\mathrm{transf}} \alpha$	$\Delta_{\mathrm{transf}} \beta$
0.0000	-0.269	-0.360	-0.250	0.47	0.0000	-0.269	-0.368	-0.246	0.54
0.0467		-0.333		0.25	0.1275	0.057	-0.001	0.119	0.13
0.0998	-0.010	-0.255	0.195	0.12	0.2390	0.074	0.003	0.151	0.10
0.1296	0.025	-0.220	0.237	0.12	0.3473	0.135	0.004	0.194	0.05
0.1501	0.041	-0.184	0.241		0.5194	0.123	0.016	0.241	0.01
					0.6489				
0.1822	0.049	-0.191	0.266	0.10		-	0.051		-0.05
0.1957	0.067	-0.146	0.265	0.08	0.8380	-	0.068		-0.06
0.2367	0.082	-0.125	0.279	0.06	0.9178	-	0.070		-0.06
0.2530	0.086	-0.101	0.265	0.05	1.0000	0.001	0.042	0.028	0.02
0.3375	0.105	-0.078	0.283	0.02					

0.3496	0.108	-0.054	0.271	-0.02
0.4405	0.122	-0.039	0.284	-0.01
0.5104	0.119	-0.039	0.276	-0.01
0.5479	0.110	-0.069	0.284	
0.6593	0.131	-0.006	0.277	-0.03
0.7567	0.071	-0.004	0.152	-0.02
0.8461	0.090	0.008	0.181	0.00
0.9029	0.063	-0.004	0.133	-0.01
1.0000	0.001	-0.004	-0.020	0.02
